# Evaluation of Candidate Measures for Home-Based Screening of Sleep Disordered Breathing in Taiwanese Bus Drivers

**DOI:** 10.3390/s140508126

**Published:** 2014-05-05

**Authors:** Hua Ting, Ren-Jing Huang, Ching-Hsiang Lai, Shen-Wen Chang, Ai-Hui Chung, Teng-Yao Kuo, Ching-Haur Chang, Tung-Sheng Shih, Shin-Da Lee

**Affiliations:** 1 Department of Physical Medicine and Rehabilitation, Chung-Shan Medical University Hospital, Chung-Shan Medical University, Taichung 40201, Taiwan; E-Mail: huating@csmu.edu.tw; 2 Center of Sleep Medicine, Chung-Shan Medical University Hospital, Chung-Shan Medical University, Taichung 40245, Taiwan; E-Mails: huangrenjing@yahoo.com.tw (R.-J.H.); csha499@csh.org.tw (S.-W.C.); csha368@csh.org.tw (A.-H.C.); 3 Institute of Medicine, Chung Shan Medical University, Taichung 40201, Taiwan; 4 Department of Medical Image and Radiological Science, Chung-Shan Medical University, Taichung 40201, Taiwan; 5 Department of Medical Informatics, Chung Shan Medical University, Taichung 40201, Taiwan; E-Mail: liay@csmu.edu.tw; 6 PhD Program of Mechanical and Aeronautical Engineering, Feng Chia University; Taichung 40724, Taiwan; E-Mail: troyguo@yahoo.com.tw; 7 Department of Photonics and Communication Engineering, Asia University, Taichung 41354, Taiwan; E-Mail: chchang@asia.edu.tw; 8 Institute of Labor Policy and Occupational Safety and Health, Ministry of Labor Affairs, Executive Yuan, Taipei 22143, Taiwan; 9 Department of Public Health, College of Public Health, China Medical University, Taichung 40402, Taiwan; 10 School of Rehabilitation Medicine, Shanghai University of TCM, Shanghai 201203, China; 11 Department of Physical Therapy, Graduate Institute of Rehabilitation Science, China Medical University, Taichung 40202, Taiwan; 12 Department of Healthcare Administration, Asia University, Taichung 41354, Taiwan

**Keywords:** diagnostic techniques and procedures, polysomnography, oximetry, obstructive sleep apnea

## Abstract

*Background*: Sleepiness-at-the-wheel has been identified as a major cause of highway accidents. The aim of our study is identifying the candidate measures for home-based screening of sleep disordered breathing in Taiwanese bus drivers, instead of polysomnography. *Methods*: Overnight polysomnography accompanied with simultaneous measurements of alternative screening devices (pulse oximetry, ApneaLink, and Actigraphy), heart rate variability, wake-up systolic blood pressure and questionnaires were completed by 151 eligible participants who were long-haul bus drivers with a duty period of more than 12 h a day and duty shifting. *Results*: 63.6% of professional bus drivers were diagnosed as having sleep disordered breathing and had a higher body mass index, neck circumference, systolic blood pressure, arousal index and desaturation index than those professional bus drivers without evidence of sleep disordered breathing. Simple home-based candidate measures: (1) Pulse oximetry, oxygen-desaturation indices by ≥3% and 4% (*r* = 0.87∼0.92); (2) Pulse oximetry, pulse-rising indices by ≥7% and 8% from a baseline (*r* = 0.61∼0.89); and (3) ApneaLink airflow detection, apnea-hypopnea indices (*r* = 0.70∼0.70), based on recording-time or Actigraphy-corrected total sleep time were all significantly correlated with, and had high agreement with, corresponding polysomnographic apnea-hypopnea indices [(1) 94.5%∼96.6%, (2) 93.8%∼97.2%, (3) 91.1%∼91.3%, respectively]. Conversely, no validities of SDB screening were found in the multi-variables apnea prediction questionnaire, Epworth Sleepiness Scale, night-sleep heart rate variability, wake-up systolic blood pressure and anthropometric variables. *Conclusions*: The indices of pulse oximetry and apnea flow detection are eligible criteria for home-based screening of sleep disordered breathing, specifically for professional drivers.

## 1. Introduction

There is an increasing accumulation of evidence to indicate that sleepiness-at-the-wheel is a major cause of traffic accidents [1,2], a situation which places a huge monetary and personal cost on society [3]. Professional long-haul bus drivers, compared with short-haul drivers, are the major group involved in this issue. Furthermore, sleep disordered breathing (SDB) in long-haul bus or truck drivers has been proven to be substantially associated with accidents [4–6]. The sleep quality of professional drivers at home was investigated by a simple device measuring snoring, heart rate, O_2_ saturation and posture change. Hui *et al.* found that 68.6% of their participants were snorers and 41% were SDB sufferers [7]. The high prevalence of pathological sleep disorders might be one of the consequences of the particular lifestyle of long-haul drivers or truck drivers, which is characterized by rare exercise, long term sleep deficiency and day-night shift working [8].

To date, overnight laboratory-based polysomnography (PSG or psg) has been regarded as the “gold standard” for the diagnosis of SDB [9], which is mainly quantified by the apnea-hypopnea index (AHI) [10]. However, the PSG study is laborious, cumbersome, expensive, technically difficult and not easily accessible. Therefore, candidate measures for home-based screening of SDB are required as substitutes for PSG.

Various investigative procedures, such as pulse oximetry, multi-variables apnea prediction questionnaire, air flow detection, actigraphy, and heart rate variability may be candidate measures for evaluating SDB or sleep problems. An overnight O_2_ saturation measurement via pulse oximetry (PO or po) has good reliability for dichotomizing participants as high and low risk for SDB [11,12]. However, low sensitivity of pulse oximetry has been reported in screening SDB patients in previous studies. Multi-variables Apnea Prediction Questionnaire (MAP) [13] and Epworth Sleepiness Scale (ESS) have often been used in questionnaires to evaluate the severity of SDB. A respiratory flow-derived signal might be reliable at home, although lacking neurological signals and analysis [14]. Moreover, an air flow detection ApneaLink (ALK) with a single channel for measuring breathing flow through nasal probes, has proved to be trustworthy in diagnosing SDB [15]. The total sleep time can be easily estimated by using Actigraphy (ACT or _ACT_) [16], and when a laboratory PSG study is not available this strategy might improve the diagnosis of SDB. However, a recent study has shown that ACT could underestimate wake time (WT), and overestimate total sleep time (TST), compared with PSG [17]. Thereafter, ACT was recommended in patients with primary insomnia or circadian rhythm disorders [18], rather than with SDB [19]. There is still doubt about the reliability of PO and air flow detection alone or combined with ACT in screening groups of various age, gender, and profession[20], and with underlying diseases [20]. As a consequence of repetitive arousal, some particular signals could at least partially reflect the sympathetic responses at arousing, such as heart rate variability. Theoretically, the sympathetic activity related signals could be seen as the surrogates of arousals [21]. Nevertheless, they are nonspecific for SDB but also appear in other sleep problems, such as spontaneous arousal, periodic limb movement, and bruxism, *etc* [22].

Heart rate variability parameters (HRVp), day or night, might represent accurate and inexpensive screening measures for clinically suspected SDB patients [23]. Furthermore, BMI, life styles or neck circumference might also be useful for predicting SDB in Asians [24].

The inappropriate sleep behavior of long-haul bus drivers is obviously linked to public safety issues. The aim of this study is to evaluate candidate measures for a home-based screening of sleep disordered breathing in long-haul bus drivers by measuring various parameters from different devices or methods, such as PO, ALK, ACT, MAP Questionnaire, ESS, HRVp, and BMI. We hypothesize that the combination of PO and flow detection corrected by ACT might be reliable tools for quantifying the AHI as a screening tool for SDB.

## 2. Methods

### 2.1. Subjects

The participants in the present study were all long-haul bus drivers, who were enrolled from November 2007 to October 2008 from major cities in Taiwan. Their duty period, more than 12 h a day, was shifted by the rule of “2 h cyclic shift day after day” to avoid interpersonal unfair arguing. The inclusion criteria were those long-haul bus drivers who were regular drivers, had not quit driving for the sake of health problems during the previous six months, and were willing to join our study. Exclusion criteria included acute diseases, acute physical injuries and mental problems one month before the current study, TST in PSG study (TSTpsg) less than three hours, female drivers, or incomplete polysomnographic data. One hundred and fifty-one male bus drivers were found to have eligible AHIpsg data. This study was a cross-sectional study and approved by the Institutional Review Board of the China Medical University Hospital, Taichung, Taiwan (CMUH IRB No. DMR98-IRB-183). All the participants were informed of the procedures and aims of this investigation, and completed consent forms before commencement.

### 2.2. Polysomnography (PSG)

This twelve-channel computerized polysomnography study (Rembrandt, Medcare, Amsterdam, The Netherlands) was performed in our sleep laboratory, simultaneously recording breathing and electrocardiography. Two channel EEGs (C3/A2, C4/A1), electro-oculograms, and submental electromyogram recordings were all determinants for sleep staging. All signals were processed in the conventional way, *i.e.*, a 30 s period as an epoch [10]. Signals recorded with pulse oximetry, oro-nasal flow sensor (pressure sensors), and chest/abdominal respiratory effort sensors were used to quantify the rate of abnormal breathing. The sleep stage, apnea and hypopnea were decided according to the criteria. Regular V_2_ ECG signals were analyzed automatically by commercial available software (HRVp, see below).

AHIpsg, the ranking standard for SDB severity, was defined as the sum of apnea and hypopnea events per hour during sleep [10,25]. Arousals were defined as episodes lasting ≥3 s (American Sleep Disorders Association) in which there was a return of α EEG activity associated with a discernible increase in electromyogram activities [26].

The parameters for the PSG study include recording time (rt; *i.e.*, the time period between lights-off and lights-on), wake time (WTpsg), TSTpsg, sleep latency (time to fall asleep after lights off), sleep efficiency (equal to total sleep time/recording-time × 100%), apnea-hypopnea index (AHIpsg), lowest O_2_ saturation (SaO_2_), duration of SaO_2_<90%, the percentage of rapid eye movement sleep (Stage REM%); and the percentages of Stage N1, N2, and N3 in non-REM sleep.

### 2.3. Anthropometric Parameters, Questionnaires and Blood Pressure

In a previous study [13], researchers collected data related to self-reported questionnaires about various symptoms of sleep apnea, and other sleep disorders, as a screening tool for sleep apnea including MAP and ESS. Multi-variables Apnea Prediction Questionnaire (MAP) and Epworth Sleepiness Scale (ESS) have often been used in questionnaires to evaluate the severity of SDB. A MAP Questionnaire is a method for modifying the prediction for sleep apnea based on information of age, gender and body mass index (BMI). The Epworth Sleepiness Scale (ESS) is a short questionnaire intended to measure daytime sleepiness. A lifestyle questionnaire included questions about habits in smoking, alcohol consumption, betel nut usage, energy-stimulating drink taking, exercise habit, regular workload and vigilance at the wheel. Betel nut is often chewed like tobacco in Taiwan, with a similar narcotic effect. Where, “Never”, “Rare”, “Occasionally”, “Usually”, and “Always” were defined as: never, 1–3 times/month, 1–2 times/week, 3–5 times/week and every day, respectively. Anthropometric parameters included body weight, height, neck circumference, waist circumference, hip circumference and their derivatives such as BMI [= body weight in kg divided by height in m^2^ (kg/m^2^)], height-normalized neck circumference, and waist/ hip ratio.

Blood pressure was taken non-invasively with an automatic device (Philips V24E) through a tourniquet worn on the right forearm, with the subject recumbent, for around 15 min before lights-off at PSG study and after lights-on the next morning. Mean arterial blood pressure was determined as =1/3 systolic blood pressure +2/3 diastolic blood pressure.

### 2.4. Portable Pulse Oximetry (PO)

Simultaneously with the PSG study, portable pulse oximetry (Pulsox-300i; Minolta, Osaka, Japan) recorded the changes of SaO_2_ and heart rate via a fingertip probe and a forearm-located recording box. From off-line data processing with company-provided software, this device reports various parameters such as O_2_ desaturation indices (ODI) and pulse rise indices (PRI), for a number of events per hour of recording-time (-rt), at 3%-O2-desaturation (ODI3po) or 4%-O2-desaturation (ODI4po); and pulse rise indices ≥7% (PRI7) or 8% (PRI8) from the baseline, respectively.

### 2.5. ApneaLink (ALK, or -alk)

ALK (ResMed Corporation, Poway, CA, USA) is a screening device which uses nasal probes to monitor breathing airflow. With a sampling rate of 100 Hz, and a 16 bit data processing system with 15 MB of in built-in memory, this battery-operated, chest-located recording box can record signals continuously for 10 h. With the company-provided software, the AHI, based on rt (AHIalk-rt), could be obtained automatically by data processing. Using ALK's original settings, apnea and hypopnea were defined as a breath flow of 10% and a 50%∼10% decline from a baseline for ≥10 s, respectively. They both should be associated with a ≥4% O_2_ saturation decline.

### 2.6. Actigraphy

An actigraphy device (Actiwatch; Neurotechnology Ltd., Cambridge, UK) [20] was worn on the wrist of the non-dominant hand. The maximal sampling frequency was set at 32 Hz to allow data to be stored in 1 min epochs. When the actiwatch is moved a bar-shaped accelerometer flexes, producing a voltage in the sensor. The degree and force of flexing evoke the voltage, which is thereafter translated into activity counts. The arousal threshold in ACT was set at an integrated activity count of 40 movements within each epoch. The actigraphy-corrected total-sleep-time (ACT) was assessed using the algorithm supplied by Sleepwatch Analysis Software for Windows. From which the WT estimated by ACT (WT_ACT_) was available.

### 2.7. Heart Rate Variability Parameters (HRVp)

The HRVp were calculated by time-domain analysis with commercialized software (Somnologica 3.1.2, Embla, Denver, CO, USA). To perform the analysis, each QRS complex from V_2_ ECG recording of PSG studies was identified, and the length between each QRS complex (*i.e.*, RR intervals) was calculated. Only normal-to-normal beats (NN) were considered for analysis. Several parameters describing the differences between RR intervals were calculated [23]: the square root of the mean of the sum of the squares of differences between adjacent NN intervals (RMSSD), standard deviations (SD) of NN intervals (SDNN), SD of the averages of NN intervals in all 5-minute segments of the recording (SDANN), and mean of the SD of all NN intervals for all consecutive 5-minute segments of the recording (SDNN index).

### 2.8. Protocol

At around 8:00 to 9:00 pm, each participant, on their own schedule, attended our sleep center. Prior to the PSG examination, the anthropometric variables, questionnaires such as ESS, MAP and Lifestyle were completed. During overnight PSG examination, portable pulse Oximetry (PO), ApneaLink (ALK), and actigraphy were recorded simultaneously in all participants, except the original settings of PSG measures. Heart Rate Variability Parameters (HRVp) were analyzed from ECG recordings of the PSG examination.

### 2.9. Analysis Steps

As a gold standard for SDB severity ranking, AHIpsg = 5 and 15 events/hr were represented as thresholds of Non-SDB (AHI < 5), Mild (15 > AHI ≥ 5), and Moderate/Severe (AHI ≥ 15) SDB. While the WT in the PSG study (WTpsg) was determined by qualified sleep technicians and specialists; the WT of actigraphy (WT_ACT_) was calculated off-line automatically with the Sleepwatch Analysis Software. When WTpsg or WT_ACT_ is subtracted from the common rt, TSTpsg or TST_ACT_ is obtained for each. Thereafter, the total “quasi breathing events” of PO or ALK measurement were numerators divided by rt or TST_ACT_, (episodic events/ hr) and available as follows: Based on either recording-time (-rt) or actigraphy-corrected total-sleep-time (ACT), the O2-desaturation indices of ≥3% and 4% (ODI3, ODI4) and pulse-rising indices of ≥7% and 8% (PRI7and PRI8) from baseline were detected with pulse oximetry (PO), while apnea-hypopnea indices were measured with ALK (AHIalk). The apnea-hypopnea indices measured with PSG are presented as AHIpsg.

In the first step the participants were partitioned into three subgroups of SDB (Non-SDB, Mild, and Moderate/Severe) by the aforementioned two thresholds (*i.e.*, two cutting points of 5 and 15 events/h in AHIpsg value). Among these subgroups, the differences between athropometric parameters, blood pressures, questionnaires and variables of PSG study were tested by one-way ANOVA, Kruskal-Wallis test, Mann-Whitney tests or Chi-Square test. The variables, when differing significantly among subgroups, would be seen as “eligible parameters A”, which would be further investigated by Receiver Operating Characteristic (ROC) curve analysis.

All pulse oximetry (PO) and ALK related rt or ACT time-based parameters (also known as eligible parameters B) were tested for similarities and agreements with the corresponding AHIpsg by Pearson's correlation and Bland-Altman plotting analyses. Moreover, all eligible parameters A and B, based on 5 or 15 events/h of AHIpsg as a SDB diagnosis threshold, were examined by ROC curve analysis to show corresponding appropriate selected points, areas under curve (AUC), and values of sensitivity and specificity. The level of AUC, sensitivity, or specificity for the discriminatory ability of each indicator was defined as “Good”, “Very Good” or “Excellent” when their values were 0.71–0.80, 0.81–0.90, or >0.90, respectively; otherwise, “Not Good”.

Software SPSS 11.0 was used for all statistical data analysis. The significant difference was defined as *p* values being less than 0.05.

## 3. Results

The lifestyle questionnaire, age, BMI, neck circumference, WHR, blood pressures, and sleep problems of these 151 drivers are shown in Tables 1 and 2. The percentages of “current” plus “experienced” smoking, alcohol and betel nut were 64%, 21% and 26%, respectively. Of all eligible subjects, 36% or 30% “always” took tea or coffee, whereas less than 30% had exercise habits. Working situations in past year showed 68% at the wheel for more than 10 h, and 26% off duty for less than 8 h each day. Moreover, 28% “usually” or “always” experienced feeling tired at the wheel but were not able to stop for a break, while 17% felt exhausted to the point of almost falling asleep while driving.

Of these professional bus drivers, 63.6% were diagnosed as having SDB (AHI > 5). The subjects (*n* = 151) were partitioned into three subgroups depending on the severity of SDB: Non-SDB (AHI < 5), Mild (15 > AHI > 5), and Moderate/Severe (AHI > 15) (Table 2).

There were no differences in age, wake-up diastolic or mean blood pressures, or MAP and ESS among subgroups. However, neck circumferences, height-normalized neck circumference and waist/hip ratio in “Mild” and “Moderate/Severe” were larger than those in “Non-SDB”. “Moderate/Severe” had a higher value of BMI than “Non-SDB” and “Mild”. Furthermore, the “Moderate/Severe” had a higher wake-up systolic blood pressure than “Non-SDB”. In the PSG study; sleep latency, TSTpsg and sleep efficiency were not different among subgroups. In sleep architecture, ‘Non-SDB’ had a less Stage N1% than “Mild” and “Moderate/Severe”, whereas no differences were noted among subgroups in Stage N2%, slow wave sleep% (*i.e.*, Stage N3%) or Stage REM%. Additionally, the “Mild” and “Moderate/Severe” groups had higher arousal indices than “Non-SDB”; whereas “Moderate/Severe” had a higher arousal index than “Mild”. The lowest SaO_2_, duration of SaO_2_ < 90% and O_2_ Desaturation Index (*i.e.*, the events/hr of O_2_ saturation decremented ≥4% from the wakefulness baseline data) in “Moderate/Severe” were worse than the other two subgroups, whereas those in ‘Mild’ were also worse than in “Non-SDB” (Table 2).

All subjects were divided into two groups by using five or 15 events/hr of AHIpsg as cutting points (Table 3). The anthropometric parameters (neck circumference, height-normalized neck circumference, BMI and waist/hip ratio), wake-up systolic pressure, ODI3po-rt, ODI4po-rt, PRI7po-rt and PRI8po-rt were higher in the “<5” group than in the “≥5” group as well as being higher in “<15” group than in “≥15” group. By contrast, the HRVp such as SDNN, SDNN Index, RMSSD and SDANN were not different between two groups, no matter which cutting point was used.

**Figure 1.**
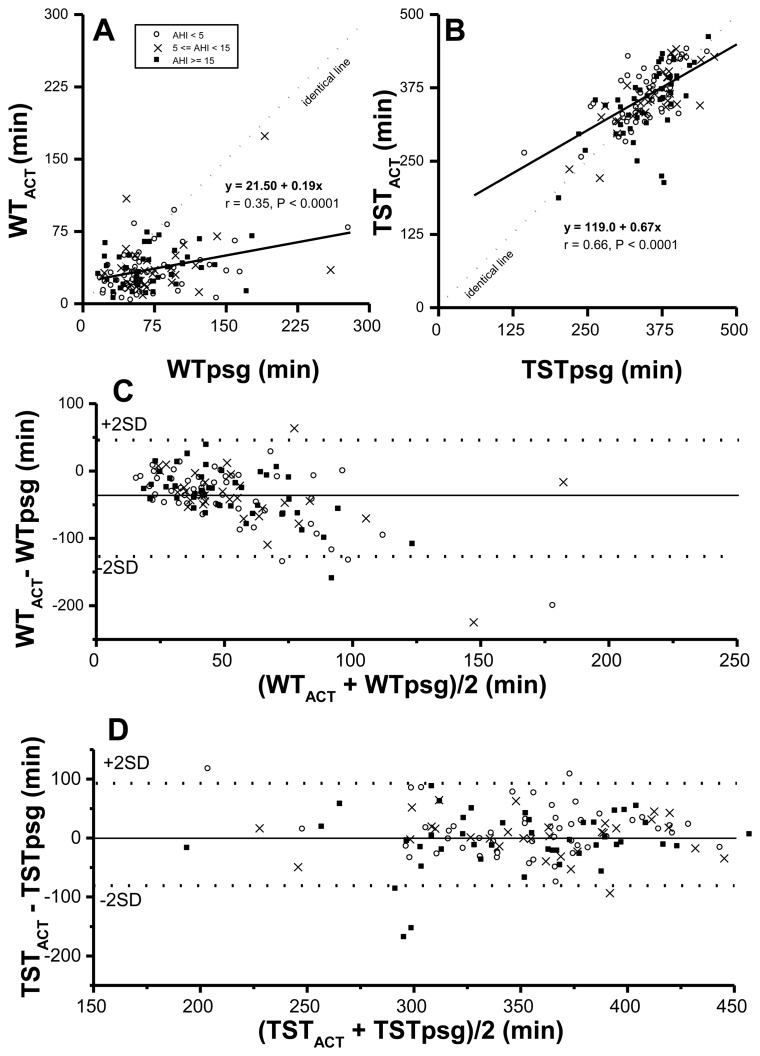
Pearson's correlation coefficients (**A**,**B**) and Bland-Altman plots (**C**,**D**) between the wake time (WT) (A,C) or total sleep time (TST) (B,D) of a polysomnography (psg) study and corresponding data obtained from actigraphy (ACT). In Bland-Altman plots, the solid line represents mean difference whereas the dotted lines represent former values ± 2 standard deviations.

Both WT and TST corrected by actigraphy-corrected total sleep time (ACT), *vs.* rt-based, might be closer to those in the PSG study. Therefore, correlations and agreements between PSG time based parameters and their corresponding ACT time-based ones were examined first. Although WT_ACT_ correlated significantly with WTpsg (*p* < 0.0001) (Figure 1A. r value was not quite as high (*r* = 0.35). By formula TST = rt-WT, derived TST_ACT_ was significantly correlated with TSTpsg (*p* < 0.0001, *r* = 0.66) (Figure 1B). Further, the value of WT_ACT_ was lower than that of its corresponding WTpsg, reflecting ACT underestimated wakeful time, no matter how severe SDB was. Moreover, by Bland-Altman plotting, 95.3% in WT_ACT_ *vs.* WTpsg (Figure 1C; Table 4) and 95.2% in TST_ACT_ *vs.* TSTpsg (Figure 1D; Table 4) were within agreement range.

**Figure 2.**
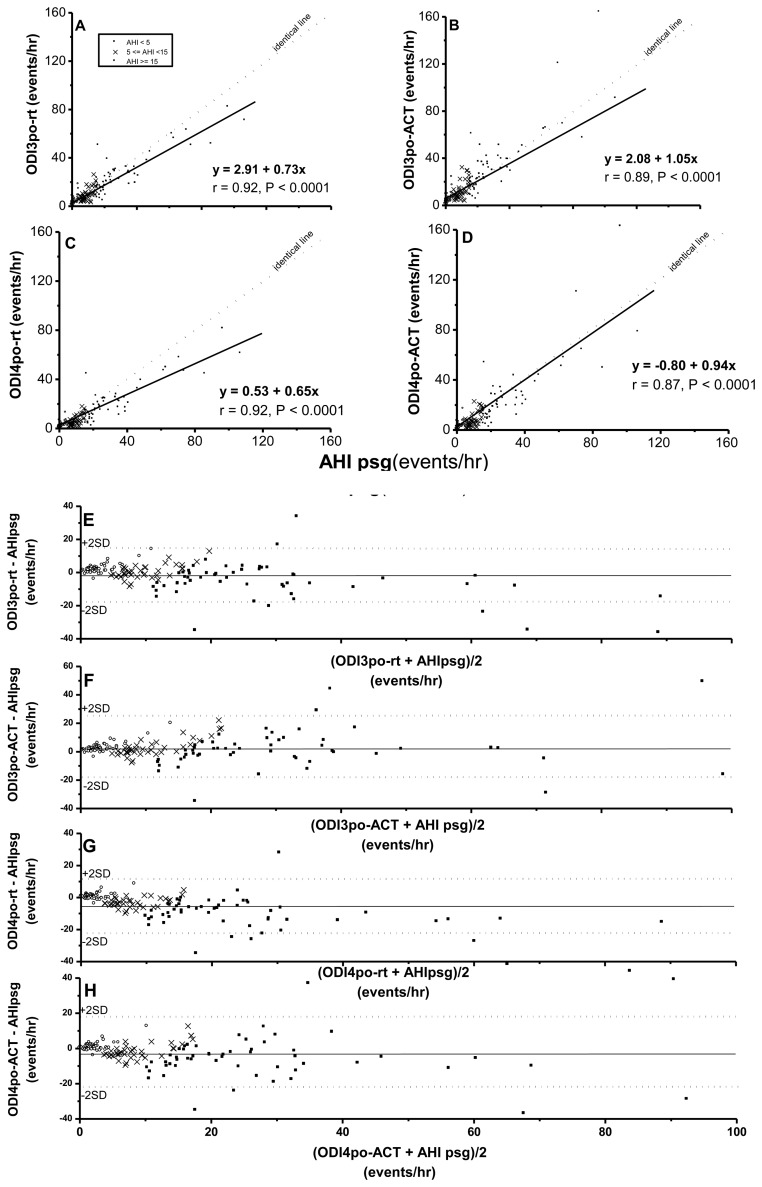
Pearson's correlation coefficients (**A**–**D**) and Bland-Aliman plots (**E**–**H**) between the apnea-hypopnea index (AHI) from polysomnography (psg) and 3% (A,B,E,F), 4% (C,D,G,H) O_2_ desaturation index from portable pulse oxymetry measurements (po) on recording-time (rt) (A,C,E,G) or Actigraphy-corrected total-sleep-time (_ACT_) base (B,D,F,H). In the Bland-Altman plots, the solid line represents mean difference whereas the dotted lines represent former values ± 2 standard deviations. A,B,C,D: same scales; X axis of E, F, G, H: same scales.

While ODI3po-rt, ODI3po-_ACT_, ODI4po-rt, and ODI4po-_ACT_ were all significantly correlated with AHIpsg (Figure 2), based on record time, ODI3po-rt and ODI4po-rt tended to be underestimated over increasing values of corresponding AHIpsg (slopes of regression lines were 0.73 and 0.65, respectively). In contrast, the values of ODI3po-_ACT_ and ODI4po-_ACT_, TST_ACT_, were closer to values of corresponding AHIpsg (slopes of regression lines were 1.05 and 0.94, respectively). By Bland-Altman plotting, 94.5%, 96.6%, 95.9%, and 94.5% in ODI3po-rt, ODI3po-_ACT_, ODI4po-rt, and ODI4po-_ACT_, respectively *vs.* AHIpsg (Figure 2E–H; Table 4) were shown within agreement ranges.

**Figure 3.**
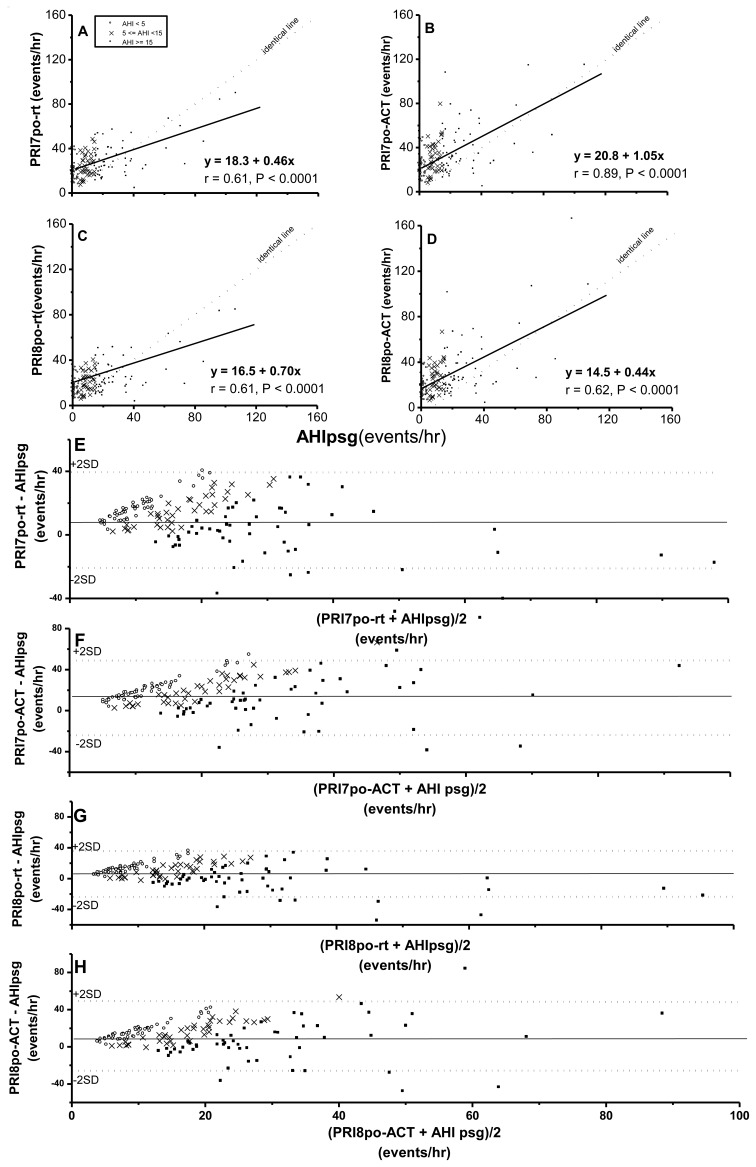
Pearson's correlation coefficients (**A**–**D**) and Bland-Aliman plots (**E**–**H**) between the apnea-hypopnea index (AHI) from polysomnography (psg) and 7% (A,B,E,F), 8% (C,D,G,H) pulse rise index from portable pulse oximetry measurements (po) on recording-time (rt) (A,C,E,G) or actigraphy-corrected total-sleep-time (ACT) base (B,D,F,H). In the Bland-Altman plots, the solid line represents mean difference whereas the dotted lines represent former values ± 2 standard deviations. A,B,C,D: same scales; X axis of E, F, G, H: same scales.

Pulse rise indices (PRI7po-rt, PRI7po-_ACT_, PRI8po-rt and PRI8po-_ACT_) were all significantly correlated with AHIpsg (Figure 3A–D). It was noteworthy that at an AHIpsg <30 events/h, the above four derivatives by Pulse oximetry were significantly overestimated based the values of AHIpsg. By Bland-Altman plotting for agreement with AHIpsg, 97.2%, 95.8%, 95.1%, and 93.8% were shown within this range in PRI7po-rt, PRI7po-_ACT_, PRI8po-rt and PRI8po-_ACT_, respectively (Figure 3E–H; Table 4).

**Figure 4.**
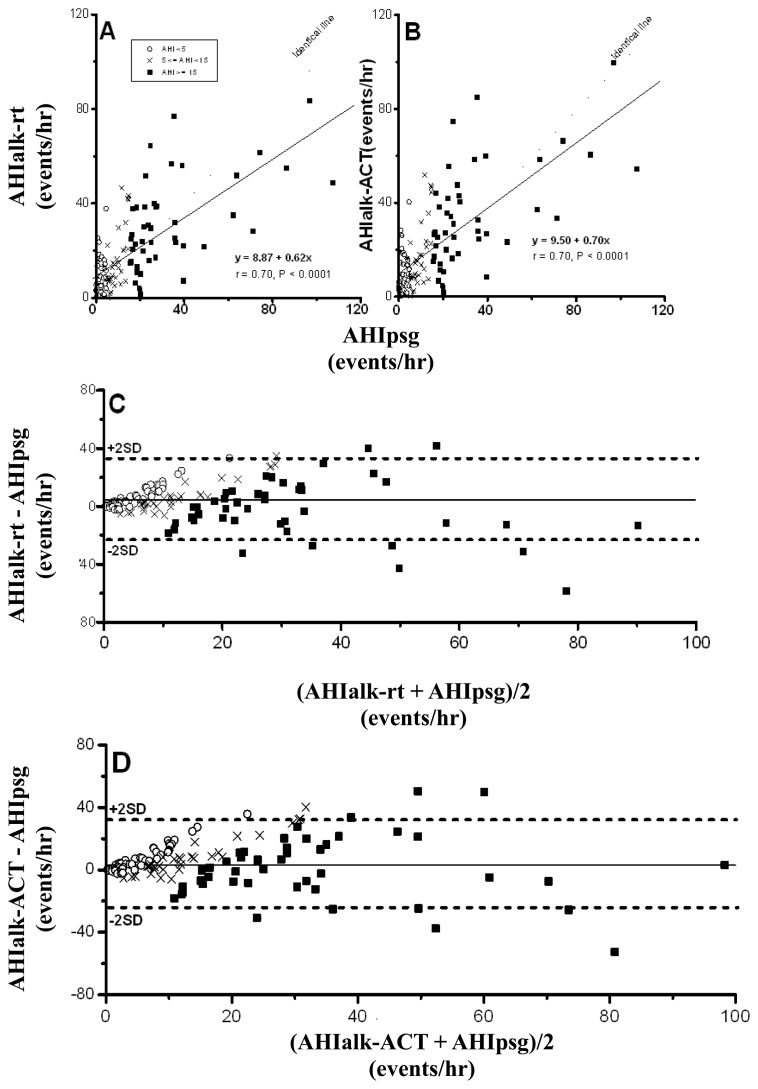
Pearson's correlation coefficients (**A**,**B**) and Bland-Altman plots (**C**,**D**) between the apnea-hypopnea index (AHI) of polysomnography (psg) and those of Apnealink (ALK) either on (A) recording-time (rt) or on (B) Actigraphy-corrected total-sleep-time (ACT). In the Bland-Altman plots, the solid line represents mean difference whereas the dotted lines represent former values ± 2 standard deviations.

Because a nasal probe is the only pathway for the detection of breathing flow in ALK, adequate signals from twenty-four eligible drivers (16%) were not available for automatic analysis due to breathing, at least partially, via the mouth. AHIalk-rt and AHIalk-_ACT_, calculated from the remaining 127 drivers, were significantly correlated with AHIpsg (Figure 4A,B), and Bland-Altman plotting testing with an AHIpsg, of 92.1% and 91.3% in AHIalk-rt and AHIalk-_ACT_, respectively was within the agreement range (Figure 4C,D; Table 4).

**Figure 5.**
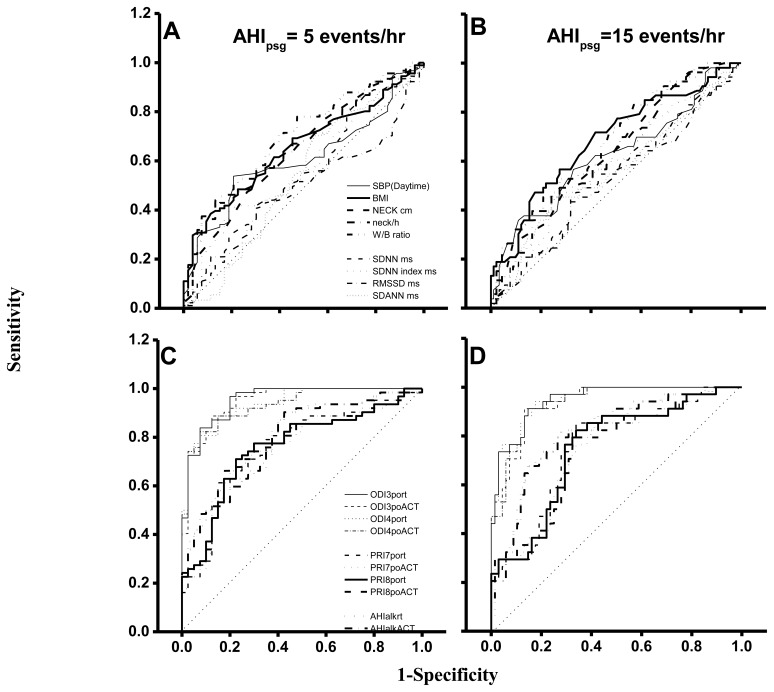
Using apnea-hypopnea indices (AHI) from polysomnography (psg) threshold values of 5 (**A**,**C**) and 15 (**B**,**D**) events/hour, the representative ROC curves of waking systolic blood pressures, anthropometric or heart rate variability parameters (A,B); and 3%, 4% Oxygen desaturation index (ODI3, ODI4) or 7% or 8% pulse rise index (PRI7, PRI8) from portable pulse oximetry measurements (po) and the apnea-hypopnea index (AHI) from ApneaLink (alk), on recording-time (rt) or Actigraphy-corrected total-sleep-time (ACT) (C,D). A, B, C, D: same scales.

In an ROC curve analysis based on AHIpsg = 5 or 15 events/hr as the threshold (Table 5; Figure 5), the AUC, sensitivity and specificity of wake-up systolic blood pressure, BMI, neck circumference, height-normalized neck circumference and waist hip ratio were 0.61∼0.69, 0.47∼0.63, and 0.64∼0.79 (AHIpsg = 5 events/h); or 0.62∼0.68, 0.62∼0.83, and 0.43∼0.58 (AHIpsg = 15 events/h), respectively.

Again, AUC, sensitivities and specificities in HRVp (such as SDNN, SDNN index, RMSSD, and SDANN) were 0.49∼0.57, 0.42∼0.70, 0.45∼0.77; or 0.52∼0.58, 0.51∼0.66, 0.41∼0.56, on the threshold of AHIpsg = 5 or 15 events/h, respectively. These results indicate that the above anthropometric parameters and HRVp were Not Good. On the contrary, ODI3po and ODI4po based on the recording time (-rt) or Actigraphy-corrected total-sleep-time (ACT) as the TST were all Excellent screening variables (sensitivity and specificity shown in Table 5). In addition, it was noteworthy that the best selected points of ODI3po-rt and ODI4po-rt were far lower in value than their corresponding AHIpsg thresholds (3.9 and 2.0 *vs.* AHIpsg = 5; 12.5 and 6.5 *vs.* AHIpsg = 15 events/hr as thresholds in ODI3po-rt; and ODI4po-rt, respectively). TST adjusted by ACT (TST_ACT_) might actually improve the similarities between ODI3po or ODI4po (*i.e.*, ODI3po-_ACT_ or ODI4po-_ACT_) and AHIpsg by narrowing the gaps between AHIpsg and selected points (from 3.9 to 4.6 or from 2.0 to 2.8 *vs.* 5 events/hr; and from 12.5 to 15.6 or from 6.5 to 9.2 *vs.*15 events/hr of AHIpsg, from ODI3po-rt to -_ACT_ and from ODI4po-rt to -_ACT_, respectively). In discriminating ability for SDB, PRI7po-rt, PRI7po-_ACT_, PRI8po-rt and PRI8po-_ACT_ were all just “Good”. Interestingly, the appropriate selected points in PRI7po-rt, PRI7po-_ACT_, PRI8po-rt and PRI8po-_ACT_ were higher in value than those of AHIpsg thresholds (e.g., 19.7 *vs.* 5 and 21.4 *vs.* 15 events/h of AHIpsg, respectively in PRI8po-_ACT_).

The values of AUC, sensitivity and specificity of ROC curve analysis in AHIalk-rt and AHIalk-_ACT_ with thresholds set on AHIpsg = 5; or 15 events/h, are shown in Table 5 and Figure 5. As screening variables both AHIalk-rt and AHIalk-_ACT_ were reaching “Good” or “Excellent” levels in SDB discrimination. However, the selective points were higher in value *vs.* the corresponding AHIpsg = 5 events/h, as the threshold. ALK might become invalid for recording attenuated breath flow if the subjects did not breathe only via the nasal pathway. Comparison among polysomnographic AHI, Apnealink automatic AHI, and manual-scored AHI in non-SDB (AHI < 5), mild SDB(AHI:5-14.9), moderate SDB (AHI:15-29.9), and severe SDB (AHI > 30) were shown in Table 6. AHI based on ApneaLink device calculated manually were closer to polysomnographic AHI when comparing AHI based on ApneaLink device calculated automatically. AHI based on ApneaLink device calculated automatically was not precise enough especially in non-SDB and mild-SDB.

The pulse oximetry and apnea flow detection might be eligible criteria and cost less for home-based screening of sleep disordered breathing, specifically for professional bus drivers. The cost of Portable Pulse Oximetry was US$1000 and the cost of ApneaLink was US$2500 based on online invoice. The overnight polysomnography cost $200 per night in Taiwan and cost much more in USA. If we exam all 1000 drivers during five years, the overnight polysomnographies totally cost US$200,000 for 1000 times. If we screen all 1000 professional bus drivers via Portable Pulse Oximetry and ApneaLink, about 300 professional bus drivers with AHI-ApneaLink >15 need to be rechecked through overnight PSG study. Total cost for screening 1000 professional drivers by using home-based screening Portable Pulse Oximetry and ApneaLink as well as 300 drivers with AHI-ApneaLink > 15 rechecking by polysomnography will cost US$83,500. In sum, the costs per person in polysomnography versus home-based Pulse Oximetry & ApneaLink are US$200 versus US$83.5.

## 4. Discussion

The current study realizes that in screening long-haul bus drivers for absence of SDB, or for moderate to severe SDB, pulse oximetry is quite reliable, in terms of using O_2_ desaturation indices (ODI) or pulse rise indices (PRI) as the indictors. Airflow detection, even applied alone, might still be an acceptable metric if the subjects could be certain of breathing only through nasal pathways during night sleep. It might be noteworthy that the similarities of pulse oximetry measures with AHIpsg would be improved if Actigraphy-corrected total-sleep-time (ACT) corrected for total sleep time. In these reliable measures the proper cutting points should be re-selected when corresponding to certain AHIpsg thresholds. By contrast, the overnight Heart Rate Variability Parameters (HRVp), Epworth Sleepiness Scale (ESS), Multi-variables Apnea Prediction Questionnaire (MAP), or anthropometric parameters are not sufficiently valid to be independent screening tools or candidate measures.

In previous studies, questionnaires [13,27], nocturnal pulse oximetry [11,27], daytime nasal resistance[28], a nasal flow sensors and/or actigraphy have been developed, but the validities of the various combinations are not clear. Moreover, some preliminary studies have indicated that the validities were adequate in screening for SDB by using an overnight sleep- and daytime-HRVp [23] or a combination of peripheral vasotonography, O_2_ saturation and actigraphy [29] as a simple screening alternative. However, most studies, in investigating validities of screen alternatives, recruited clinical patients rather than professional drivers. To our knowledge, this might be one of the few studies enrolling long-haul bus drivers in examining validities of the potential SDB screening alternatives for polysomnography.

The “autonomic arousals”[30] are phenomena of several autonomic responses, such as elevated blood pressure, increased heart rate and skin vasoconstriction. These arousals appeared at various arousing stimuli, no matter whether cortical activities were evoked, or not. A previous study has indicated that the severity of SDB could be ranked by these physiological responses. However, wake-up systolic blood pressure in the present study was not a good candidate measures for discriminating SDB. Our previous study disclosed that post- to pre-night sleep systolic blood pressure change, instead of wake-up or pre-sleep systolic blood pressure, was associated with SDB severity in clinical patients. Even so, this warrants further studies to clarify the association between blood pressure and SDB severity in professional drivers.

Theoretically, the pulse rise index (PRI) and HRVp may be considered as heart rate surrogates reflecting autonomic arousals. However, in the current study no overnight HRVp was found to be as “Good” as the pulse rise index (PRI7po and PRI8po) detected from pulse oximetry in screening for SDB. On the other hand, Guilleminault *et al.* [31] have indicated that in clinical SDB patients, the more frequent that breathing events occurred, the greater the heart rate fluctuations would be reflected. Furthermore, other previous research [23] has shown that time-domain heart rate variability indices were eligible candidate measures in screening SDB patients, with a threshold of 10 events/h in AHIpsg. Although it is hard to explain the inconsistency of HRVp between different studies or with the pulse rise index in the current research, the eligibility of this metric to perform as an SDB screening measure may offer some insights into the special lifestyle features of our professional bus drivers.

As a consequence of long-term tedious working periods, sleep restriction, circadian rhythm derangement, plus prevalent smoking, alcohol consumption, betel nut usage, the taking of stimulating drinks, and the rarity of regular exercise, the drivers' lifestyle might elevate night-sleep HRVp's values so diffusedly as to attenuate their sensitivity to specific SDB episodes. This hypothesis might be supported by the fact that values of SDNN, SDNN index, RMSSD and SDANN at sleep time in our individuals with AHIpsg ≤ 5 events/h (*i.e.*, “non-SDB” group) were greater than those of clinical SDB patients with AHIpsg ≤ 10 events/ h in Roche *et al.*'s study [23]. In the current study, we might also note that HRVp relatively reflected a persistent autonomic situation; whereas PRI7po or PRI8po, reflected an episodic autonomic response. The former might indicate a sustained status with various amplitudes of heart rate fluctuations as frequent autonomic arousals over night-sleep in our subjects, regardless of the severity of SDB. In contrast, the latter might be a threshold setting for episodic heart rate rising (*i.e.*, ≥7% or 8% heart rate elevation against baseline values), reflecting autonomic arousals with this feature evoked somehow concomitant with SDB events. Nevertheless, those autonomic arousals, of high amplitude heart rate rising, appeared more frequently than the disordered breathing episodes in the vast majority of our subjects with AHIpsg ≤ 30 events/h (Figure 3A–D). However, whether these phenomena are specific for the drivers merits further studies.

The questionnaires of ESS and MAP are not eligible for SDB screening in the present study. Three factors might be the causes, as follows: (1) Short-term naps occurred from time to time in drivers' waiting rooms, leading to daytime-hypersomnolence; (2) A certain new physiological adaptation might develop to cope with their special lifestyle; (3) To avoid disturbing their family's sleep, the drivers of irregular duty-periods often slept alone, weakening the discriminating power of the questionnaires. Furthermore, and in contrast to Caucasians, in Asians the BMI may not be as significant as some craniofacial differences vulnerable to SDB [32]. It seems plausible that the discriminative ability of certain tools to screen SDB might be affected by various physiological characteristics of the targeted occupational groups.

In previous studies [11,12,20], the validity of pulse oximetry in screening potential SDB patients has not always been encouraging in terms of values of sensitivity and specificity, especially when AHIpsg threshold are set to less than 25. There is still debate about the threshold for an abnormal ODI in screening for SDB. Moreover, a recent study used the criteria of O_2_ desaturation 4% ≥ 10 events/h or O_2_ desaturation 3% ≥15 events/h to screen workers, and found 100% of workers with AHI ≥ 5 and 88.9% workers with AHI ≥ 20. Although the ROC analysis, sensitivity and specificity were not available, the authors claimed pulse oximetry was a good screening tool for SDB patients. In contrast, pulse oximetry in the present study has been found to be a reliable device for screening for SDB. Poor sensitivities and specificities and lack of discriminating ability were found in anthropometric parameters, blood pressure, sleep questionnaires, and heart rate variability variables in screening SDB from the professional bus drivers. In contrast, using ODI3po-rt = 3.9 and ODI4po-rt = 2.0 events/hr as cutting points for screening in subjects with AHIpsg ≥ 5 events/h (Table 5), can provide excellent sensitivities (0.97 and 0.94, respectively) and good specificities (0.80 and 0.70, respectively). A similar situation was seen in ODI3po-rt or ODI4po-rt screening at a threshold of AHIpsg = 15 events/h. A linear regression analysis of ODI4po-rt against AHIpsg provided a correlation coefficient r = 0.92 (Figure 2C) and an intercept value of 0.53, quite close to zero, which indicates a good correlation. A discrepancy between the present study and previous ones might be caused by the following reasons: (1) Different definitions for hypopnea: in the current study, 10%–50% baseline breathing flow is associated with ≥4% O_2_ desaturation from the baseline, regardless of association with arousals or previous arousals; (2) Corresponding cutting points' selection: At ROC analysis undertaking, we selected the proper cutting points for these alternative candidate measures, according to the screening principle of a sensitivity preference plus an acceptable specificity, instead of using cutting points with the same values as the given AHIpsg threshold in previous studies, which possibly ignores the reality that total sleep time is always less than recording time; (3) The subjects of the current study were all long-haul bus drivers, whose lifestyles might favor the likelihood of O_2_ desaturation of 4% or more from baseline, when sleep breathing flow is less than half the baseline for more than 10s. In other words, these exhausted subjects potentially could not be aroused till moderately reduced breath flow was accompanied with a deeper O_2_ desaturation. Previous or current smokers putatively had a worsening ventilatory/perfusion matching in the pulmonary capillary-alveolar environment, [33] which might bias the subjects to arterial O_2_ desaturation when breathing flow diminished.

Surprisingly, in the current study, subjects using ALK to screen for SDB appeared to be not as reliable as in a previous study [15] (AUC were 0.79 *vs.* 0.86 and 0.82 *vs.* 0.98 in the current study *vs.* the previous study on the thresholds of 5 and 15 events/hr in AHIpsg values, respectively). Additionally, in 24 drivers (16%), ALK breath flow signals were too shallow to be recognized by autonomic analytic software. However, they were probably similar in terms of demographic data and values of AHIpsg if compared to overall subjects. After reciprocally checking simultaneous breathing signals from PSG and ALK, this fault might be the result of signal amplitude waning at the mouth opening during breathing. However, there is little previous research where this shortcoming in ALK's breath flow measurement via a nasal probe has been realized. Craniofacial abnormalities [32] and allergic rhinitis [34], quite prevalent in subtropical Asians, may increase the resistance of the nasal airway and lead to mouth breathing, particularly during sleep. Accordingly, we putatively predict that equipped with an oronasal flow probe, ALK will improve the reliability of screening SDB patients.

Actigraphy has not been used for routine screening SDB [19]. Nevertheless, it seems reasonable that total sleep time would be predicted more precisely by subtracting movement period from recording time. By using a simplified device for simultaneously measuring oronasal breath flow, respiratory efforts and fingertip oxygen saturation. Elbaz *et al.* confirmed that actigraphy could narrow the gap between total sleep time measured by alternative tools [16]. In the current study, the wake time via actigraphy was significantly lower in value than that via PSG, although they were still correlated with each other (*r* = 0.35, *p* < 0.0001). This phenomenon might indicate that the exhausted drivers, even at cortical arousal (*i.e.*, EEG arousal) from sleep, could not move their body sufficiently to be sensed by actigraphy, resulting in waking time periods far shorter than those calculated from EEG criteria. Sensitivity, specificity and AUC among ODI3po, ODI4po, PRI7po, or PRI8po or the correction of actigraphy for total sleep time correction were not different (Table 5). This feature was consistent with previous studies.[35,36] However, it did not mean that total sleep time correction was unusable, but verified again the fact that an overestimation of TST, before being corrected, at least plays in part a role in an inappropriate setting of values of ODIs in screening for SDB corresponding to the AHIpsg threshold.

In conclusions, anthropometric parameters, waking blood pressure, sleep questionnaires, and heart rate variability during night sleep have a lack of discriminating ability in screening for SDB in professional bus drivers. The indices of pulse oximetry and apnea flow detection are appropriate to design the simplest home-based candidate measures to screen for SDB, specifically for professional drivers. The reliability of ALK might be improved if a sensitive oro-nasal flow probe were to be developed. ODI4po, ODI3po, PRI7po or PRI8po on recording a time base could potentially be good candidate measures for screening professional bus drivers for SBD, when proper corresponding cutting points are taken into consideration. Actigraphy might offer a more precise total sleep time and further improve the similarities of the above pulse oximetric parameters with the ‘gold standard’ AHI from PSG. In brief, ODI3po and ODI4po are Excellent; PRI7po and PRI8po are Good; AHIalk, in conditions of appropriate signal catching is Excellent to Very Good; while the other aforementioned parameters are Not Good candidate measures for screening professional drivers for SDB.

### Limitation

There are some limitations in the current study. To date, most sleep specialists have reached a consensus that AHI by lab-based overnight polysomnography (*i.e.*, AHIpsg) appears to be a “gold standard” in judging SDB severity. However, there are some limitations in PSG examination and in all measurement, such as the first-night effect of PSG. There are some biases due to the first-night effect. In addition, although the pulse oximetry and apnea flow detection are eligible criteria for home-based screening of sleep disordered breathing, specifically for professional drivers, AHI based on ApneaLink device detected automatically was still not precise enough especially in non-SDB and mild-SDB. Nevertheless, due to the high cost of PSG examination and a limited availability of sleep PSG labs, it would be difficult to provide screening for SDB for most professional drivers. Although alternative, practical, screening tools appear to be urgently needed, screening tools might still miss catch some real SDB cases.

## Figures and Tables

**Table 1.** The life style and driving features.

	**Current**	**Experienced**	**Never**	**Rare**	**Occasionally**	**Usually**	**Always**
**Smoking**	13 (8)	87 (56)	54 (35)				
**Alcohol**	6 (4)	26 (17)	121 (79)				
**Betel Nut Taking**	13 (8)	28 (18)	112 (73)				
**Tea Drinking**			29 (19)	25 (16)	44 (29)		56 (36)
**Coffee Drinking**			36 (24)	25 (17)	45 (30)		45 (30)
**Regular Exercise**	44 (29)		110 (71)				
**Bus driving more than 10 hours a day**			6 (4)	14 (9)	29 (19)	61 (40)	43 (28)
**Off-duty period less than eight hours a day**			36 (24)	34 (22)	43 (28)	24 (16)	16 (10)
**Hard to stop driving, even felt tired**			41 (27)	36 (23)	34 (22)	28 (18)	15 (10)
**Exhausted at driving caused by a break shortage**			35 (23)	47 (31)	46 (30)	20 (13)	6 (4)

n (%); The definitions of “Experienced”: not current behavior, “Never”:never so far, “Rare”:1–3 times/month, “Occasionally”:1–2 times/week, “Usually”: 3–5 times/week, and “Always”: every day. Abbreviation: n and %, number of and percentage of these alleged drivers who had respond the related-questionnaires.

**Table 2.** The body and sleep characteristics among three subgroups of sleep disordered breathing.

	**Total**	**(1) Non-SDB**	**(2) Mild SDB**	**(3) Moderate/ Severe SDB**	*p value*	*post hoc*
AHIpsg, events/hr	15.2 ± 18.5	2.1 ± 1.4	9.4 ± 3.1	32.0 ± 20.8		
n=	151	55	39	57		
Age, yrs	42.3 ± 6.6	41.8 ± 6.3	41.3 ± 6.9	43.8 ± 6.8	0.2360	
Body Mass Index, kg/m^2 §^	26.7 ± 3.7	25.2 ± 2.5	26.4 ± 3.6	28.5 ± 4.2	0.0001	(3 > 1), (3 > 2)
Neck Circumference, cm	39.1 ± 2.7	38.0 ± 2.7	39.1 ± 2.74	40.0 ± 2.6	0.0012	(2 > 1), (3 > 1)
Neck Circumference (Normalized), cm/m	23.2 ± 1.7	22.8 ± 1.6	23.2 ± 1.8	24.0 ± 1.6	0.0012	(2 > 1), (3 > 1)
Waist/ Hip Ratio ^§^	0.94 ± 0.10	0.90 ± 0.06	0.96 ± 0.17	0.95 ± 0.06	<0.00001	(2 > 1), (3 > 1)
Systolic Blood Pressure, mm Hg	124 ± 14	119.3 ± 11.5	123.4 ± 14.0	128.0 ± 14.5	0.0044	(3 > 1)
Diastolic Blood Pressure, mm Hg	81 ± 11	78.9 ± 9.8	80.5 ± 10.8	82.0 ± 11.4	0.3755	
Mean Blood Pressure, mm Hg	95 ± 11	92.4 ± 10.0	94.8 ± 11.5	97.3 ± 11.2	0.0959	
Multivariable Apnea Prediction	14.3 ± 9.3	12.5 ± 8.2	15.9 ± 10.5	15.3 ± 9.3	0.1442	
Epworth Sleepiness Scale	5.7 ± 3.6	5.5 ± 4.0	6.0 ± 3.4	5.9 ± 3.5	0.6496	
Sleep Latency, min	16.0 ± 17.6	18.0 ± 16.6	17.2 ± 24.3	13.3 ± 12.0	0.2985	
Total Sleep Time, min	352 ± 52	350.1 ± 49.7	351.9 ± 54.0	353.9 ± 52.0	0.9272	
Sleep Efficiency, % ^§^	82.4 ± 10.0	82.7 ± 10.4	81.7 ± 10.8	82.8 ± 9.0	0.8927	
Non REM N1, % ^§^	7.5 ± 5.0	6.0 ± 3.2	7.8 ± 4.3	8.9 ± 6.4	0.0082	(2 > 1), (3 > 1)
N2, %	56.2 ± 10.5	57.8 ± 9.8	54.3 ± 10.2	55.9 ± 11.1	0.1582	
N3, %	7.5 ± 8.7	6.8 ± 6.9	7.0 ± 7.1	6.3 ± 7.3	0.5783	
REM, %	16.3 ± 6.4	16.6 ± 6.5	17.0 ± 6.2	15.7 ± 6.6	0.5961	
Arousal Index, events/hr ^§^	31.7 ± 17.2	24.8 ± 12.1	29.5 ± 13.3	40.2 ± 20.0	<0.00001	(2 > 1), (3 > 1), (3 > 2)
Lowest Oxygen Saturation, % ^§^	79.1 ± 11.6	86.7 ± 4.9	80.7 ± 7.2	70.6 ± 13.1	<0.00001	(1 > 2), (1 > 3), (2 > 3)
Duration of SaO_2_<90%, min ^§^	24.9 ± 51.0	3.0 ± 7.3	12.8 ± 28.1	54.1 ± 70.3	<0.00001	(2 > 1), (3 > 1), (3 > 2)
O_2_ Desaturations Index, events/hr* ^§^	15.1 ± 16.4	2.7 ± 2.0	10.6 ± 5.8	30.0 ± 17.4	<0.00001	(2 > 1), (3 > 1), (3 > 2)

Abbreviations: SDB, sleep disordered breathing; Non-SDB (AHI < 5), Mild (15 > AHI ≥ 5), and Moderate/Severe (AHI ≥ 15); n, number of subjects; AHIpsg, apnea hypopnea index by polysomnographic study; REM, rapid eye movement stage *By definition, O_2_ Desaturations Index in this table is identical to ODI4psg TST psg in Table 4. ^§^Kruskal-Wallis test; post hoc: Mann-Whitney U test Significant differences within two subgroups shown in ( ).

**Table 3.** The differences between two subgroups of AHIpsg < 5 and ≥5 or < 15 and ≥15 events/hour.

	**AHIpsg (events/hr)**			**AHIpsg (events/hr)**		
	**< 5**	**≥ 5**	***p value*^a^**	**<15**	**≥15**	***p value*^a^**
**n =**	55	96		94	57	
**AHIpsg, events/hr**	2.0 ± 1.4	22.7 ± 19.6		5.1 ± 4.3	31.8 ± 20.9	
**Systolic Blood Pressure, mm Hg**	120 ± 12	126 ± 14	0.0076	121 ± 13	128 ± 15	0.0048
**Body Mass Index, kg/m^2^**	25.3 ± 2.4	27.6 ± 4.1	0.0006	25.8 ± 3.0	28.5 ± 4.2	0.0001
**Neck Circumference, cm**	38.2 ± 2.6	39.7 ± 2.7	0.0024	38.6 ± 2.7	40.0 ± 2.6	0.0040
**Neck Circumference (Normalized), cm/m**	22.5 ± 1.4	23.6 ± 1.7	0.0002	22.8 ± 1.6	23.8 ± 1.6	0.0009
**Waist/ Hip Ratio**	0.91 ± 0.05	0.95 ± 0.11	<0.0001	0.93 ± 0.12	0.95 ± 0.06	0.0003
**SDNN, ms**	98 ± 29	104 ± 36	0.4093	99 ± 29	105 ± 40	0.5231
**SDNN Index, ms**	73 ± 23	81 ± 35	0.1637	75 ± 26	84 ± 39	0.1357
**RMSSD, ms**	68 ± 33	73 ± 58	0.8712	68 ± 33	77 ± 71	0.7365
**SDANN, ms**	83 ± 117	73 ± 54	0.5297	74 ± 91	81 ± 67	0.3069
**ODI3po-rt, events/hr**	3.8 ± 3.2	20.0 ± 14.8	<0.0001	10.0 ± 5.2	26.2 ± 14.8	<0.0001
**ODI3po-_ACT_, events/hr**	4.7 ± 4.1	26.0± 22.1	<0.0001	12.6± 7.1	35.1.± 22.0	<0.0001
**ODI4po-rt, events/hr**	2.3 ± 2.10	15.2 ± 13.2	<0.0001	6.4 ± 3.7	21.2± 13.2	<0.0001
**ODI4po-_ACT_, events/hr**	2.8 ± 2.7	20.0 ± 20.3	<0.0001	8.2 ± 5.1	28.1± 20.2	<0.0001
**PRI7po-rt, events/hr**	18.6 ± 8.3	29.2± 14.1	<0.0001	25.0 ± 9.8	32.1± 14.1	<0.0001
**PRI7po-_ACT_, events/hr**	22.5 ± 11.2	37.9± 22.7	<0.0001	31.2± 13.6	42.7± 22.6	<0.0001
**PRI8po-rt, events/hr**	15.0 ± 7.0	24.8 ± 13.3	<0.0001	20.8 ± 8.6	27.6 ±13.3	<0.0001
**PRI8po-_ACT_, events/hr**	15.3 ± 9.4	32.5± 21.5	<0.0001	26.0 ± 11.7	37.1 ± 21.5	<0.0001
**AHIalk-rt, events/hr**	7.9± 7.9	24.9 ± 18.6	<0.0001	11.0 ± 10.8	31.2 ± 19.5	<0.0001
**AHIalk--_ACT_, events/hr**	8.6 ± 8.6	27.3± 20.8	<0.0001	12.1± 11.8	34.3± 22.3	<0.0001

**A**bbreviations: AHI, apnea & hypopnea index; psg, data got from the polysomnographic studies; n, number of subjects; SDNN, standard deviation of all NN intervals; SDNN Index, mean of the standard deviations of all NN intervals for all 5-minute segments of the entire recording; RMSSD, the square root of the mean of the sum of the squares of differences between adjacent NN intervals; SDANN, standard deviation of the averages of NN intervals in all 5-minute segments of the entire recording; ODI3 or ODI4, the index (events/hr) of 3% or 4% and more oxygen desaturation from baseline values; PRI7 or PRI8, the index (events/hr) of 7% or 8% and more pulse rising from baseline values; po, data got from the potable pulse oximetry. ^a^ Mann-Whitney test.

**Table 4.** The values of parameters of Bland-Altman plots' agreement analysis.

		**n =**	**Mean Difference ± 2(SD)**	**Underestimated n (%)**	**Overestimated n (%)**	**Agreement n (%)**
**WTpsg**						
	**WT_ACT_**	127	−40.9 ± 2× 19.5	5 (3.9)	1 (0.8)	121 (95.3)
**TSTpsg**						
	**TST_ACT_**	125	2.5 ± 2 × 43.3	4 (3.2)	2 (1.6)	119 (95.2)
**AHIpsg**						
	**ODI3po-rt**	146	−0.8 ± 2 × 4.1	6 (4.1)	2 (1.4)	138 (94.5)
	**ODI3po-_ACT_**	146	1.2 ± 2 × 5.4	2 (1.4)	3 (2.1)	141 (96.6)
	**ODI4po-rt**	146	−2.5 ± 2 × 4.4	5 (3.4)	1 (0.7)	140 (95.9)
	**ODI4po-_ACT_**	146	−1.0 ± 2 × 5.2	4 (2.7)	4 (2.7)	138 (94.5)
	**PRI7po-rt**	144	5.0 ± 2 × 7.6	3 (2.1)	1 (0.7)	140 (97.2)
	**PRI7po-_ACT_**	144	8.5 ± 2 × 9.4	3 (2.1)	3 (2.1)	138 (95.8)
	**PRI8po-rt**	144	2.9 ± 2 × 4.7	7 (4.9)	0 (0.0)	137 (95.1)
	**PRI8po-_ACT_**	144	5.9 ± 2 × 9.0	6 (4.2)	3 (2.1)	135 (93.8)
	**AHIalk-rt**	127	3.2 ± 2 × 14.5	6 (4.7)	4 (3.1)	117 (92.1)
	**AHIalk-_ACT_**	127	5.0 ± 2 × 15	6 (4.7)	5 (3.9)	116 (91.3)

Abbreviations: WT, waking time; TST, total sleeping time; AHI, apnea & hypopnea index; psg, data got from the polysomnographic studies; ACT, time period corrected by actinography; rt, total recording time period;alk, data got from ApneaLink device; ODI3 or ODI4,the index (events/hr) of 3% or 4% and more oxygen desaturation from baseline values;PRI7 or PRI8, the index (events/hr) of 7% or8% and more pulse rising from baseline values;po, data got from the potable pulse oximetry; n, number of subjects; SD, standard deviation.

**Table 5.** The most appropriate selected point, area under the curve, sensitivity and specificity by ROC curve.

	**AHIpsg =5**				**AHIpsg =15**			
	**Selected Point**	**AUC**	**Sensitivity**	**Specificity**	**Selected Point**	**AUC**	**Sensitivity**	**Specificity**
**SBP, mm Hg**	127	0.61	0.54	0.79	124.5	0.62	0.62	0.58
**Body Mass Index, kg/m^2^**	26.1	0.65	0.58	0.64	26	0.68	0.72	0.58
**Neck Circumference, cm**	39.8	0.64	0.47	0.74	38.8	0.64	0.66	0.48
**Neck Circumference (normalized), cm/m**	23.3	0.67	0.56	0.70	22.5	0.65	0.72	0.45
**Waist/ Hip Ratio**	0.9	0.69	0.63	0.66	0.9	0.66	0.83	0.43
**SDNN, ms**	106	0.54	0.44	0.60	87.0	0.54	0.66	0.41
**SDNN Index, ms**	86.5	0.57	0.42	0.77	70.5	0.58	0.60	0.52
**RMSSD, ms**	71	0.49	0.42	0.70	62.5	0.52	0.51	0.54
**SDANN, ms**	49.5	0.53	0.70	0.45	60.5	0.56	0.51	0.56
**ODI3po-rt, events/hr**	3.9	0.95	0.97	0.80	12.5	0.95	0.91	0.85
**ODI3po-_ACT_, events/hr**	4.6	0.95	0.97	0.78	15.6	0.94	0.91	0.87
**ODI4po-rt, events/hr**	2.0	0.94	0.94	0.70	6.5	0.95	0.94	0.82
**ODI4po-_ACT_, events/hr**	2.8	0.93	0.92	0.73	9.2	0.94	0.91	0.85
**PRI7po-rt, events/hr**	18.6	0.76	0.81	0.63	22.0	0.75	0.82	0.68
**PRI7po-_ACT_, events/hr**	24.1	0.76	0.74	0.70	27.1	0.74	0.74	0.68
**PRI8po-rt, events/hr**	15.9	0.76	0.77	0.70	17.9	0.75	0.82	0.66
**PRI8po-_ACT_, events/hr**	19.7	0.76	0.74	0.70	21.4	0.74	0.79	0.66
**AHIalk-rt, events/hr**	9.2	0.79	0.73	0.65	14.6	0.82	0.82	0.72
**AHIalk-_ACT_, events/hr**	8.2	0.79	0.82	0.63	14.8	0.81	0.82	0.69

Abbreviations: SBP, Systolic Blood Pessure; SDNN, standard deviation of all NN intervals; SDNN Index, mean of the standard deviations of all NN intervals for all 5-min segments of the entire recording; RMSSD, the square root of the mean of the sum of the squares of differences between adjacent NN intervals; SDANN, standard deviation of the average of the average of NN intervals in all 5 min segments of the entire recording; ODI3 or ODI4, the index (events/hr) of 3% or 4% and more oxygen desaturation from baseline values; PRI7 or PRI8, the index (events/hr) of 7% or8% and more pulse rising from baseline values; psg, po or alk, data got from polysomnographic study, potable pulse oximetry, or Apnealink; AHI, apnea & hypopnea index; rt, recording time based data; ACT, actigraphy corrected total sleep time based data; AUC, area under curve.

**Table 6.** Comparison among polysomnographic AHI, ApneaLink automatic AHI, and manual-scored AHI.

	**Non-SDB**	**Mild SDB**	**Moderate SDB**	**Severe SDB**
AHIpsg	**<5**	**5−14.9**	**15−29.9**	**>30**
n=	20	20	20	20
AHIpsg, events/hr	0.6 ± 0.5	8.9 ± 2.6 *	18.9 ± 3.1 *^#^	50.3 ± 18.2 *^#&^
TST_psg_, min	(331 ± 38.1)	(371 ± 55.4)	(366.7 ± 50.7)	(324.2 ± 66.4)
AHI_alk-a_, events/hr	11.2 ± 10.4	15.3 ± 12.7	20.9 ± 10.7 *	39.3 ± 19.8 *^#&^
AHI_alk-m_, events/hr	1.1 ± 0.5	12.4 ± 4.9 *	20.7 ± 5.1 *^#^	34.9 ± 13.2 *^#&^
AHI_psg/rtalk_, events/hr	0.5 ± 0.4	7.7 ± 2.0 *	16.2 ± 3.1 *^#^	40.6 ± 18.4 *^#&^
rt_alk_, min	(393.7 ± 33.3)	(422.6 ± 43.4)	(437 ± 31.7)	(418 ± 35.1)

SDB = Sleep Disordered Breathing; AHI = apnea & hypopnea index; PSG = Polysomnography related values; TSTpsg = Total Sleep Time determined by polysomnography; alk = ApneaLink device related values; AHIalk-a = the values of AHI based on ApneaLink device calculated automatically; AHIalk-m = the values of AHI based on ApneaLink device calculated manually. AHIpsg/rtalk = AHIpsg relative to recording time by ApneaLink device; rtalk = recording time by ApneaLink device. * *p* < 0.05 significant difference from Non-SDB; ^#^ significant difference from Mild SDB; ^&^ significant difference from Moderate SDB.
